# Analyzing the Outcomes of COVID-19 Infection on Patients With Comorbidities: Insights From Hospital-Based Study

**DOI:** 10.7759/cureus.55358

**Published:** 2024-03-01

**Authors:** Ramya Bakthavatchalam, Sriram Bakthavatchalam, Shyamala Ravikoti, Bhaskaran Shanmukham, Kotha S Reddy, Janardhana R Pallavali, Archana Gaur, Jeganathan Geetha, Sakthivadivel Varatharajan

**Affiliations:** 1 Biochemistry, Thanjavur Medical College, Thanjavur, IND; 2 Orthopedics, Vellore Medical College and Hospital, Vellore, IND; 3 Microbiology, All India Institute of Medical Sciences, Bibinagar, Hyderabad, IND; 4 General Medicine, Melamruvathur Adhiparasakthi Institute of Medical Sciences and Research, Melmaruvathur, IND; 5 General Medicine, All India Institute of Medical Sciences, Bibinagar, Hyderabad, IND; 6 Physiology, All India Institute of Medical Sciences, Bibinagar, Hyderabad, IND; 7 General Medicine, Karpaga Vinayaga Institute of Medical sciences and Research Center, Maduranthagam, IND

**Keywords:** ischemic heart disease, hypothyroidism, mortality, comorbidity, covid-19

## Abstract

Introduction

COVID-19 exhibits a broad spectrum of clinical manifestations, ranging from asymptomatic or mild cases to severe respiratory distress and, in some instances, fatal outcomes. The pre-existing inflammatory state in the patient prior to exposure to COVID-19, which could be because of any etiology or comorbidity, has been associated with prolonged morbidity, and adverse outcomes like increased mortality have been found. This study endeavors to investigate the principal risk factors linked to the morbidity and mortality of COVID-19, such as age, gender, and co-morbidities such as hypertension, diabetes mellitus, and others.

Material and methods

Patient demographic data like age, gender, and co-morbidities like diabetes mellitus, hypertension, respiratory illness, and coronary artery diseases, cerebrovascular accident was observed. The patient clinical profile, hematological, inflammatory markers at the time of admission, and outcome were noticed. Patients were divided into two groups - patients with comorbidity and those without comorbidity.

Results

In each cohort of COVID-19 patients, comprising those with and without comorbidities, there were 145 participants. The mean age of patients without comorbidities was found to be 49.97 years, whereas the mean age of those with comorbidities was 64.35 years. Within the comorbidity group, males formed the majority, accounting for 77.2% of the cohort; in the group without comorbidity also males predominated, representing 68.3% of the participants. Hypertension was the most common co-morbidity (89.7%), followed by diabetes mellitus (39.3%), and ischemic heart disease (8.3%). The multivariate logistic regression analysis for prediction of mortality showed hypothyroidism with odds ratio (OR) of 336.26 and confidence intervals (CI) (1.19-9477.13), ischemic heart disease with OR of 320.94 (CI 3.19-3237.4) and presence greater than two co-morbidities with OR of 42.14 (CI 1.34-1325.76). Cox regression analysis showed a statistically significant hazard ratio of 0.294 in patients with greater than two co-morbidities.

Conclusion

Hypothyroidism, ischemic heart disease, and the presence of multiple comorbid conditions were associated with the severity of COVID-19 illness and mortality.

## Introduction

The novel coronavirus emergence of SARS-CoV-2 which resulted in a global pandemic named COVID-19 has significantly impacted public health, healthcare systems, and daily life worldwide. First identified in December 2019 in Wuhan, China, COVID-19 quickly spread, leading to widespread illness, extensive morbidity, and a substantial number of fatalities [1]. COVID-19 exhibits a broad spectrum of clinical manifestations, ranging from asymptomatic or mild cases to severe respiratory distress and, in some instances, fatal outcomes. In addition to the varied acute manifestations of COVID-19, some reports classify long-term COVID-19 as the persistence of symptoms, which can occur continuously or intermittently and involve multiple organ systems [2].

Pre-existing inflammatory conditions like obesity, diabetes, and coronary vascular disease in patients before COVID-19 exposure, whether due to any cause or comorbidity, have been linked to longer morbidity and negative outcomes, such as higher mortality rates. In a systematic review and meta-analysis of COVID-19 risk factors it was found to be hypertension, diabetes mellitus, cardiovascular diseases (CVDs), and prior respiratory conditions are associated with raised mortality with COVID-19 infection [3]. It is observed that previous stroke is significantly associated with higher morbidity and mortality in COVID-19 [4]. Hypothyroidism is advocated as a risk for COVID-19 infection and is also associated with significantly higher mortality [5].

This study endeavors to investigate the principal risk factors linked to the morbidity and mortality of COVID-19, elucidating the intricate interplay among individual characteristics such as age, gender, smoking, and health status, encompassing comorbidities such as hypertension, diabetes mellitus, and others. Additionally, the study explores the association of these risk factors with the presentation of symptoms and the severity of the disease.

## Materials and methods

This retrospective observational study was carried out after obtaining Institute Ethics Committee (Human Studies) approval [MAPIMS/IEC/52/2022/210(5)2022]. The study was conducted at the tertiary care center which caters the rural areas predominantly. The data were obtained from the medical records department of the hospital from July to August 2021. Patients with COVID-19 infection confirmed by RTPCR were included. Patient demographic data like age, gender, smoking and alcohol habits, and co-morbidities like diabetes mellitus, hypertension, respiratory illness, and coronary artery diseases, cerebrovascular accident were observed. The patient’s symptoms, blood parameters like hemoglobin, total count, neutrophil-lymphocyte ratio (NLR), platelet count, erythrocyte sedimentation rate (ESR), renal function test, and liver function tests were noted. Inflammatory markers like C-reactive protein (CRP), interleukin-6 (IL-6), procalcitonin, d-dimer, ferritin, and N-acetyl cysteine activated creatine kinase (CKNAC) at the time of admission were noted. Clinical severity was assessed by MOHFW and WHO criteria. Major complications and patient outcomes in terms of mortality were noticed. Patients were divided into two groups as patients with comorbidity and those without comorbidity.

Statistical analysis

SPSS software version 25 (IBM Corp., Armonk, NY, USA) was used for analysis. Continuous variables were represented as mean±SD. Categorical variables were represented as frequency and percentage. Continuous variables and categorical variables were analyzed by independent t-test and chi-square test respectively. Parameters with P-value of <0.25 in t-test and chi-square analysis were included in multivariable logistic regression to assess the predictors of mortality. Cox regression analysis was done for the significant parameters in multivariable logistic regression to measure the hazard ratio of variables. A P-value of ≤0.05 was considered statistically significant.

## Results

In each cohort of COVID-19 patients, comprising those with and without comorbidities, there were 145 participants. The mean age of patients without comorbidities was found to be 49.97 years, whereas the mean age of those with comorbidities was 64.35 years. Within the comorbidity group, males formed the majority, accounting for 77.2% of the cohort; in the group without comorbidity also males predominated, representing 68.3% of the participants. Hypertension was the most common co-morbidity (89.7%), followed by diabetes mellitus (39.3%), ischemic heart disease (8.3%), cerebrovascular accidents, and respiratory illness contributing the same amount (2.8%). There were 5.5% hypothyroid patients, two patients with brain tumors, and one patient with chronic kidney disease (Table 1).

**Table 1:** General characteristics of study participants

Parameter	Patient without co-morbidity (n=145)	Patient with co-morbidities (n=145)	P-value
Age in years (mean±SD)	49.97±11.77	64.35±9.45*	<0.001
Male	99 (68.3%)	112 (77.2%)	0.164
Female	46 (31.7%)	23 (22.8%)	
Smoking	4 (2.8%)	3 (2.1%)	1.000
Duration of hospitalization (days)	8.44±4.52	9.17±4.58	0.731
Co-morbidities:			
Hypertension	-	130 (89.7%)	0.000
Diabetes mellitus	-	57 (39.3%)	0.000
Ischemic heart disease	-	12 (8.3%)	0.000
Cerebrovascular accident	-	4 (2.8%)	0.122
Respiratory illness	-	4 (2.8%)	0.122
Bronchial asthma		2	
Old pulmonary tuberculosis		1	
Interstitial lung disease		1	
Chronic kidney disease	-	1 (0.7%)	1.000
Hypothyroid	-	8 (5.5%)	0.007
Brain tumor	-	2 (1.4%)	0.498

The patients with co-morbidities had significantly greater incidences of cough (56.6% vs 45.5%) and respiratory illness (51% vs 24.8%) compared to those without co-morbidities. The incidence of fever was also more but not statistically significant. Patients with comorbidity had significant critical illness. A significant difference was observed in the values of leucocyte count, NLR, CRP, and platelet count, which were greater in the co-morbid group (Tables 2, 3).

**Table 2:** Clinical profile of the study population

Parameter	Patient without co-morbidity (n=145)	Patient with co-morbidities (n=145)	P-value
Fever	75 (51.7%)	88 (60.7%)	0.155
Cough	66 (45.5%)	82 (56.6%)*	0.078
Respiratory distress	36 (24.8%)	74 (51%)*	0.000
Loss of smell & taste	1 (0.7%)	1 (0.7%)	1.000
Sore throat	5 (3.4%)	2 (1.4%)	0.447
Muscle pain	13 (9%)	8 (5.5%)	0.365
Headache	3 (2.1%)	3 (2.1%)	1.000
MoHFW criteria			0.000
Asymptomatic	28 (19.3%)	9 (6.2%)	
Mild	71 (49%)	56 (38.6%)	
Moderate	32 (22.1%)	33 (22.8%)	
Severe	14 (9.7%)	47 (32.4%)*	
WHO criteria			0.000
Asymptomatic	28 (19.3%)	9 (6.2%)	
Mild	71 (49%)	56 (38.6%)	
Moderate	32 (22.1%)	33 (22.8%)	
Severe	9 (6.2%)	15 (10.3%)	
Critical	5 (3.4%)	32 (22.1%)*	
Complications	11 (7.6%)	54 (37.2%)*	0.000
Mortality	4 (2.8%)	49 (33.8%)*	0.000

**Table 3:** Inflammatory profile and biochemical parameters of the study population CKNAC - N-acetylcysteine activated creatine kinase

Parameter	Patient without co-morbidity (n=145)	Patient with co-morbidities (n=145)	P-value
Hemoglobin (g/dl)	12.95±1.96	12.8±2.04	0.520
Total leucocyte count (cells/μL)	7340.96±3593.34	8530.97±4322.97*	0.011
Neutrophil lymphocyte ratio	3.79±5.01	7.05±7.25*	0.000
platelet count (cells/μL)	233165.52±95144.12*	261675.86±100838.07	0.014
Erythrocyte sedimentation rate (mm/hour)	34.2±19.2	37.68±22.28	0.156
C-reactive protein (mg/dl)	46.1±55.12	59.69±60.07*	0.046
Aspartate transaminase (AST) (U/L)	46.34±46.5	39.42±23.83	0.112
Alanine transaminase (ALT) (U/L)	36.26±27.57	38.07±30.79	0.598
Urea (mg/dl)	32.82±21.57	37.79±31.18	0.116
Creatinine (mg/dl)	1.16±0.79	1.55±4.5	0.298
Lactate dehydrogenase (U/L)	329.95±223.58	351.06±197.46	0.395
CKNAC (U/L)	85.4±72.9	78.3±74.5	0.412
Procalcitonin	0.19±0.6	3.86±43.8	0.315
Interleukin-6 (pg/ml)	146.37±299.94	129.84±303.98	0.642
D-Dimer (μg/ml)	1.97±7.3	2.9±12.8	0.453
Ferritin (ng/ml)	410.21±479.18	495.8±510.89	0.142

The multivariate logistic regression analysis for prediction of mortality showed hypothyroidism with odds ratio (OR) of 336.26 and confidence intervals (1.19-9477.13), ischemic heart disease with OR of 320.94 (CI 3.19-3237.4), and presence greater than two co-morbidities with OR of 42.14 (CI 1.34-1325.76) (Table 4).

**Table 4:** Multivariable logistic regression analysis for prediction of mortality MOHFW - Ministry of Health and Family Welfare, WHO - World Health Organisation

Parameters	Odds Ratio (Confidence Interval)	P-value
Age	-	0.672
Gender	-	0.533
Duration of hospitalization (days)	-	0.816
Hypertension	-	0.977
Diabetes mellitus	-	0.072
Ischemic heart disease	320.94 (3.19-3237.4)	0.014*
Hypothyroidism	336.26 (1.19-9477.13)	0.043*
Cerebrovascular accident	-	0.980
Respiratory illness	-	0.993
Single comorbidity	-	0.998
≥2 Comorbidities	42.14 (1.34-1325.76)	0.034*
Fever	-	0.633
Cough	19.58 (1.62-235.59)	0.019*
Respiratory distress	-	0.077
Total count	1 (0.99-1.0)	0.041*
Neutrophil lymphocyte ratio	-	0.981
Platelet count	-	0.880
Erythrocyte sedimentation rate	-	0.239
C reactive protein	-	0.092
Ferritin	-	0.268
Aspartate transaminase	-	0.945
Urea	-	0.073
MOHFW severity	-	0.386
WHO severity	0.002 (0.000-0.109)	0.003*

**Figure 1:**
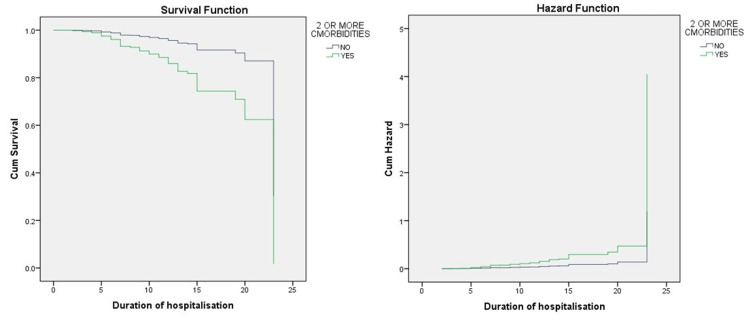
Cox proportional hazard ratio showing the relation of mortality with COVID-19 based on the presence of greater than or equal to two comorbidities

On analysis for prediction of mortality by Cox-regression analysis, the hazard ratio of hypothyroidism was 0.656, and ischemic heart disease showed a hazard ratio of 0.376, which was not statistically significant. A statistically significant hazard ratio of 0.294 was observed in patients with ≥2 co-morbidities and 2.95 was observed with WHO severity of COVID infection. (Table 5, Figure 1)

**Table 5:** Cox regression analysis for prediction of mortality

Parameters	Hazard Ratio (Confidence Interval)	P-value
Hypothyroidism	0.656 (0.094-4.593)	0.671
Ischemic heart disease	0.376 (0.113-1.250)	0.110
≥2 Comorbidities	0.294 (0.153-0.568)	0.000*
Cough	0.649 (0.364-1.155)	0.142
Total count	1.00 (1.00-1.00)	0.880
WHO severity	2.956 (2.123-4.117)	0.000*

## Discussion

Multiple comorbid conditions are attributed to the severity of morbidity and mortality of COVID-19 infection. In this study, Hypertension was the most common co-morbidity (89.7%), followed by Diabetes mellitus (39.3%), ischemic heart disease (8.3%), cerebrovascular accidents, and respiratory illness contributing the same amount (2.8%). These findings are in accordance with the published current literature [6,7].

In our study, 145 cases were included in each group of patients with comorbidity and patients without comorbidity. In either of the groups fever was the most common symptom and presentation which is followed by cough and respiratory distress. This frequency of symptoms in the presentation of COVID-19 is in accordance with previous studies, but there are good numbers of studies that differ from this finding of frequency of clinical presentations, in a study in Brazil first common symptom is dyspnea [8-10]. The variability in the frequency of symptom presentation can be attributed to several factors, including disparities in the timing of studies, ethnic demographics, and the emergence of diverse COVID-19 variants across different waves of the pandemic. Furthermore, variations in viral loads among individuals and the heightened infectivity observed during subsequent waves, such as the second wave in India, contribute to the observed differences. Notably, rapid disease progression with early onset of dyspnea during the second wave contrasts with the presentation patterns observed during the initial wave of the pandemic, highlighting the evolving nature of COVID-19 manifestations over time. In our study, while characterizing the patients for severity according to MOHFW criteria significantly greater number of patients were in the severe category in those with co-morbidity whereas according to WHO criteria, significantly greater patients were in the critical category. They also had greater complications and increased mortality compared to those without co-morbidity. These study findings align with previous studies, and it is comprehensible that the risk escalates concomitantly with the presence of comorbidities, as the overall health status of the patient deteriorates, thereby exacerbating the likelihood of increased severity and mortality rates [11].

In this study, there was a significant difference observed in the values of leucocyte count, NLR, CRP, and platelet count which were greater in the co-morbid group. These contrasts with other studies; where it is observed that reduced platelet, lymphocyte, hemoglobin, eosinophil, and basophil counts, along with elevated neutrophil count and neutrophil-lymphocyte and platelet-lymphocyte ratios, are linked to increased severity and mortality in COVID-19 infection [11]. The variation in platelet count, leukocyte count, and NLRs observed could stem from differences in inflammation levels, the severity and stage of the patient's condition upon presentation, and potentially the viral load at the time of sampling. These factors can collectively impact the hematological parameters measured and may lead to disparities in results across different cases. In our study, parameters like hemoglobin, ESR, aspartate transaminase (AST), urea, creatinine, Lactate dehydrogenase (LDH), CKNAC, procalcitonin, interleukin-6 (IL-6), d-dimer, and ferritin did not show any significant difference between the two groups of COVID-19 with comorbidities and without comorbidities. In a meta-analysis investigating the association between clinical biomarkers like CRP, procalcitonin, serum ferritin, and D-dimer with the severity of COVID-19, all parameters were associated with severe disease and poor outcomes [12]. The observed variance in inflammatory marker levels seems intricately linked to the clinical stage of disease advancement, the immunological vigor present in elderly individuals, and the inadvertent utilization of immunosuppressive agents such as dexamethasone, and prednisolone, despite lacking prescription. This unintended pharmacological modulation may have effectively dampened immune reactivity, thereby mitigating disparities between the two cohorts under investigation. In a cross-sectional study, elevated levels of CRP, IL-6, and LDH are associated with patients needing invasive mechanical ventilation indicating severe disease [13].

A meta-analysis and a systemic review which included 42 studies has observed that there is an increased risk of mortality with chronic obstructive pulmonary disease (COPD), CVD, diabetes, hypertension, obesity, cancer, acute kidney injury, and increased D-dimer [14]. Furthermore, findings from a retrospective observational study conducted in Australia by Liu et al. provide additional support for the correlation between diabetes mellitus and the severity of COVID-19 illness with the adjusted hazard ratio of 1.88 [15]. The Swedish study on COVID-19 outcomes in older adults suggests surprising findings regarding the link between comorbidities and mortality risk, highlighting the need for deeper investigation. Age-related factors like frailty and physiological changes in impaired immune response may play a significant role in influencing how chronic conditions impact COVID-19 outcomes in this population [16,17]. The absence of significant associations between certain comorbidities and mortality in the geriatric population in the Swedish study may seem surprising; it underscores the need for comprehensive and context-specific investigations to elucidate the nuanced interactions between age, comorbidities, and COVID-19 outcomes.

In our investigation, employing multivariate logistic regression analysis to predict mortality, hypothyroidism exhibited a notable odds ratio of 336.26 (95% CI: 1.19-9477.13), while ischemic heart disease displayed a substantial OR of 320.94 (95% CI: 3.19-3237.4). Additionally, the presence of more than two comorbidities demonstrated a significant OR of 42.14 (95% CI: 1.34-1325.76). These findings underscore the pronounced association of hypothyroidism, ischemic heart disease, and multiple comorbidities with mortality risk in COVID-19 patients. It’s important to note that the VIRUS Study from the COVID-19 registry did not find preexisting hypothyroidism to increase the odds of disease severity and mortality [18]. The discrepancy in findings may stem from methodological disparities, furthermore, disparities in ethnicity and sample sizes between the two studies contribute to this variance. This suggests the need for further research to understand the varying impacts of comorbidities on COVID-19 outcomes across different populations or settings. Similar to our study, Szarpak et al. found that CAD was also associated with increased severity of COVID-19 disease with OR of 2.28 (95%CI: 1.59 to 3.27) [19]. In concurrence with our study, in a large study in the UK by Chudasama et al., mortality was more than double in patients with multimorbidity (37.2% versus 17.3%) [20].

On analysis for prediction of mortality by Cox-regression analysis, the hazard ratio of hypothyroidism was 0.671, and ischemic heart disease showed a hazard ratio of 0.376, which was not statistically significant. A statistically significant hazard ratio of 0.294 was observed in patients with greater than 2 co-morbidities. These findings emphasize the complex interplay of various comorbidities in predicting mortality risk associated with COVID-19. While hypothyroidism, ischemic heart disease, and the presence of greater than two comorbidities emerged as significant predictors of mortality in our multivariate logistic regression analysis, the lack of statistical significance in the hazard ratios obtained through Cox regression analysis underscores the complexity of these associations. Nevertheless, the notable hazard ratio observed in patients with multiple comorbidities emphasizes the cumulative impact of concurrent health conditions on COVID-19 outcomes. These findings emphasize the importance of comprehensive risk assessment and tailored management strategies to mitigate mortality risk in individuals with comorbidities affected by COVID-19.

Our study was done in a single center which may limit its external validity. However, we have included an adequate sample population that will strengthen our study results.

## Conclusions

In conclusion, we have highlighted the various comorbidities among patients with COVID-19 infection with various symptoms, inflammatory profiles, and the outcome. We have found substantial associations between hypothyroidism, ischemic heart disease, and the presence of multiple comorbid conditions with the severity of COVID-19 illness and subsequent mortality.

Our study underestimates the prevalence of hypertension and diabetes mellitus as the primary comorbidities in COVID-19 infection. These findings emphasize the critical role of comprehensive risk assessment and targeted interventions for individuals with these predisposing factors to mitigate adverse outcomes in COVID-19 management.
